# Tongue Hemangioma on Dorsal Surface: A Case Report

**DOI:** 10.7759/cureus.48669

**Published:** 2023-11-11

**Authors:** Prasanna R Sonar, Aarati Panchbhai, Pooja Dhole

**Affiliations:** 1 Oral Medicine and Radiology, Sharad Pawar Dental College and Hospital, Datta Meghe Institute of Higher Education and Research, Wardha, IND; 2 Oral Medicine and Radiology, Vidarbha Youth Welfare Society's Dental College and Hospital, Amravati, IND

**Keywords:** soft tissue, radiofrequency, tongue, surgery, hemangioma

## Abstract

Benign vascular tumors known as hemangiomas usually appear in early childhood and adulthood. It rarely occurs in the oral cavity and is most frequently observed in the head and neck area. Oral hemangiomas are always clinically significant and need to be diagnosed as soon as possible and treated appropriately. Because the tongue is a highly mobile muscular organ and is, therefore, more prone to trauma and associated consequences, tongue hemangiomas pose a significant risk to patients. We are presenting a case of hemangioma on the dorsal surface of the tongue in a female patient of two years of age. The case report describes the lesion's diagnostic features and available treatment options while highlighting the importance of color Doppler ultrasonography, particularly for diagnosis and treatment.

## Introduction

Tongue hemangiomas are benign vascular tumors that are seldom encountered and can cause bleeding, pain, trouble breathing, difficulty biting, and difficulty speaking [1]. About 60% to 70% of hemangioma lesions are seen in the head and neck area. Hemangiomas are developmental vascular anomalies that are defined by a proliferative growth phase and a very slow, unavoidable regression (involution phase) [2]. A few weeks after birth, head and neck hemangiomas develop quickly [3]. Histologically, hemangiomas can be capillary, cavernous, or mixed. The most prevalent location for hemangiomas is on the skin, where 80% of lesions are solitary and 20% are bilateral. The ratio of men to women is one-third. The head and neck are the sites of more than half of the lesions. Hemangiomas are most frequently found on the cheeks, upper lip, and upper eyelids. They are rarely seen on the tongue and, when present, are typically very small. Hemangiomas often develop after birth and tend to reduce in size by about 10% per year following their proliferation phase. Treatment options for hemangiomas include surgery, corticosteroids, sclerosing agents, radiation therapy, diathermy, electrocauterization, cryosurgery, embolization, laser therapy, radiofrequency treatment, and interferon [4-6]. This report presents a case involving a two-year-old patient diagnosed with a hemangioma on the tongue and discusses the available treatment options.

## Case presentation

**Figure 1:**
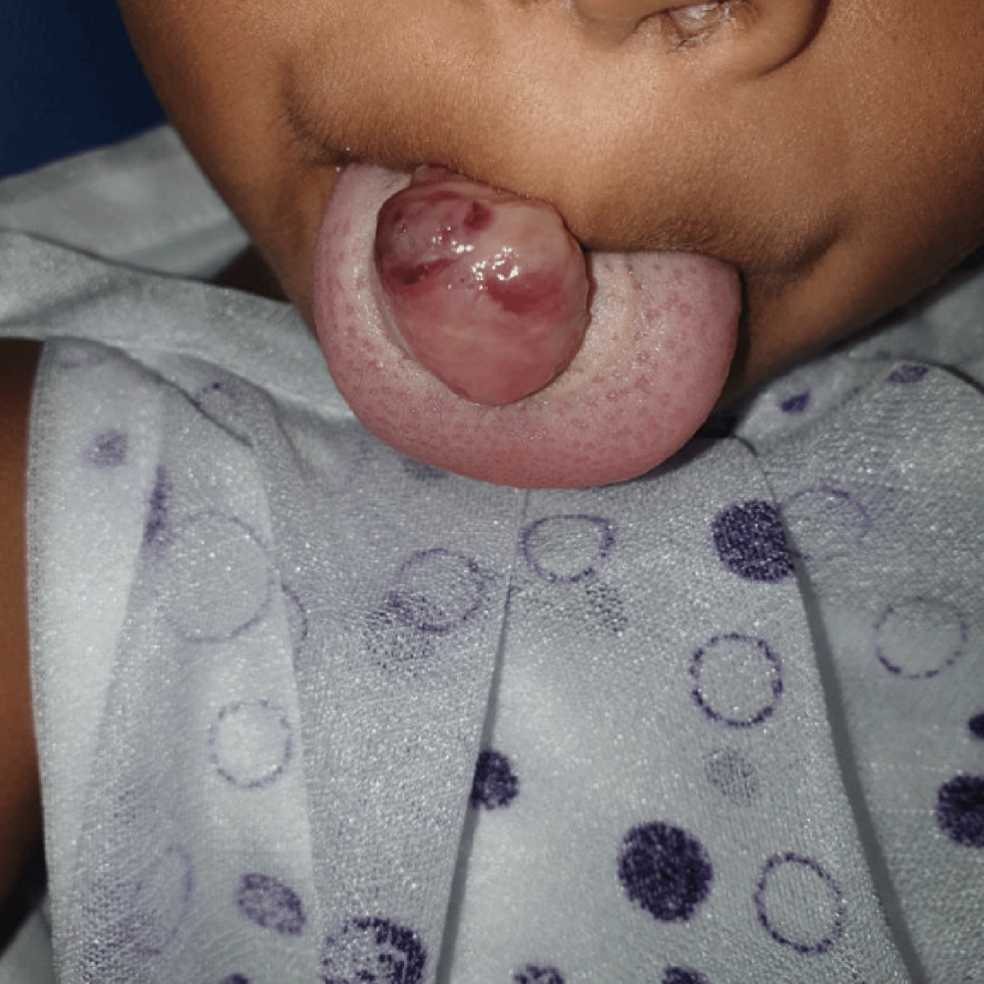
Hemangioma on dorsal surface of tongue. Image Credit: Prasanna R. Sonar

A two-year-old pediatric female patient reported at the dental hospital with a complaint of painless swelling on the dorsal surface of the tongue since birth. This painless swelling was gradually increasing in nature. The initial lesion was a bluish patch and gradually increased to a present size of approximately 3X3 cm. There was no history of bleeding or discharge from the swelling. Additionally, no history of lip biting or trauma was reported. The patient's past medical and dental histories were not significant. During the intraoral examination, a lobulated, bluish-red soft tissue swelling with a diffuse periphery was observed on the dorsal surface of the tongue, specifically in the middle one-third of the tongue, with a size of approximately 3x3 cm, as shown in Figure 1. The surface was smooth, and the margins were diffused. On palpation, tenderness was absent with a soft consistency. Fluctuation and compressibility were present. The swelling was in fixity to underlying tissue. Based on the clinical symptoms, hemangioma of the tongue was tentatively diagnosed. USG of the tongue was advised, which revealed a well-defined rounded lesion noted on the anterior aspect of the tongue, showing no vascularity. On color Doppler ultrasound, it was suggestive of hemangioma on the dorsal surface of the tongue. In this case, biopsy and fine needle aspiration cytology were not recommended. Additional diagnostic techniques, such as CTA and MRI, were recommended before deciding on a final course of treatment. However, the patient did not return for further treatment. Color Doppler ultrasonography would have been utilized to identify the feeder vessel prior to surgical resection. The planned procedure involves ligation of the feeding vessel under general anesthesia, followed by surgical excision. The excised specimen would then be sent for histopathological examination to confirm a definitive diagnosis of cavernous hemangioma.

## Discussion

Hemangiomas are known to be more common in women. The face, lips, tongue, oral mucosa, and trunk are the most often afflicted areas of the head and neck [7]. They make up about 7% of benign tumors overall. Around age one, one in ten children have hemangiomas. These lesions typically emerge during the earliest months of life rather than at birth. 70-90% of them appear within the first one to four weeks. Of these lesions, 20% have more than one lesion, and 80% have single lesions. Hemangiomas have three distinct stages: growth, stability, and involution. About age 5, 70% of cavernous hemangiomas invert, followed by the remaining 10% between ages 10 and 12. The involution stage progresses more quickly the earlier it begins. Although they typically happen infrequently, autosomal dominant family transmission has also been documented [8]. The hemangioma can range in size from a few millimeters to several centimeters and is characterized by a soft mass that is sessile or pedunculated, smooth or lobulated [9-10]. Hemangiomas are often very red and may blanch when pressure is applied. Larger hemangiomas can pose challenges for chewing [11-12]. Deeper lesions typically have a dome shape and exhibit either a typical red or blue surface tone; they rarely blanch upon pressure. In contrast, superficial hemangiomas are often lobulated and tend to blanch when pressed with a finger. An arteriovenous hemangioma, also known as an arteriovenous aneurysm, A-V shunt, or arterio-venous malformation, is often the lesion responsible for symptoms like a thrill, bruit, or a noticeably warmer surface. This type of malformation occurs when blood flows directly from the venous to the arterial system, avoiding the capillary beds [3]. Hemangiomas are easily diagnosed by visual inspection. To learn more about the vascularization of big hemangiomas and to determine the depth of the mass, contrast-enhanced MRI or angiography may be necessary [13]. In our case, Doppler USG was used for the same purpose. We opted for color Doppler ultrasound to identify the feeding vessel, as biopsy and fine needle aspiration cytology were not advisable. This approach was intended to aid in the surgical procedure.

Pyogenic granuloma, chronic inflammatory gingival hyperplasia, telangiectasia, and squamous cell carcinoma are among the conditions that can be seen in the differential diagnosis of hemangiomas. Pyogenic granuloma, a reactive lesion that bleeds readily, grows quickly, and is typically accompanied by inflammation and ulceration, is frequently confused with hemangiomas. Clinically, it is frequently reddish-purple, pedunculated, lobulated, and perhaps hormone-sensitive [14]. Furthermore, hemangiomas might be mistaken for face or mouth cavity lesions resembling vascular structures, such as those seen in Sturge-Weber syndrome [15]. They are frequently found in the areas where the trigeminal nerve branches are located. In these situations, ocular and brain vascular lesions might be discovered. Hemangioma's differential diagnosis should also take arteriovenous malformations into account. Hemangiomas are frequently bounded lesions that most frequently occur intraorally on the tongue, lips, and buccal mucosa. They hardly ever damage bone. An arteriovenous aneurysm, also known as an arteriovenous malformation, is most likely the lesion causing the thrill or bruit or exhibiting a noticeably warmer surface. This type of malformation involves the blood flowing directly from the venous to the arterial system, avoiding the capillary beds. Additionally, poorly defined lesions known as arteriovenous malformations can also harm bone. Because the type of vascular lesion may significantly impact treatment, an accurate diagnosis is crucial [16]. The Osler-Weber-Rendu syndrome, Sturge-Weber syndrome, and blue rubber bleb nevus syndrome are among the syndromes linked to vascular malformation. For this disorder, granuloma fasciale, insect bite, pyogenic granuloma, and angiosarcoma can be considered as differential diagnoses [3,17]. The available therapies for hemangiomas include electrocauterization, laser, embolization, radiofrequency, radiation therapy, surgery, corticosteroids, sclerosing agents, diathermy, and interferon [4-5]. Treatment is typically recommended only in certain situations, such as when there is a palpable lump, repeated bleeding, or aesthetic disfigurement. To minimize potential complications, it is advisable that surgical procedures be performed by surgeons who specialize in this area. Aggressive surgical intervention and preservation of the surrounding important structures are two things that should be avoided. Complete removal of hemangiomas can be challenging, and recurrences are common. Consequently, multistage surgery is now highly recommended. Steroids and tracheotomy openings should be utilized to treat big hemangiomas pressing on the airway. Due to systemic side effects, corticotherapy should only be used as one of the therapeutic choices in specific instances [4]. Another course of treatment is radiation therapy. Radiation therapy shrinks hemangiomas; however, it also severely atrophy the treated area's tissues, particularly the skin. In later years, it may also result in cancer. It is not favored as a result. Hemangiomas can be treated with sclerosing agents. Cryotherapy is a viable option. Still, the success rate is rather modest. It can have positive outcomes for superficial lesions. Because radiofrequency therapy does not require incisions, especially when treating shallow oral cavity hemangiomas, minimal bleeding occurs, sutures are not needed, and pain is minimized, it is a safe and successful form of treatment [18-20].

## Conclusions

The tongue, being a highly mobile muscular organ, is more susceptible to trauma and its associated consequences. Conversely, oral soft tissue hemangiomas can resemble other lesions both histologically and clinically. In the case mentioned, the female child had a hemangioma on the tongue, which posed a significant risk for trauma and its related consequences. Early diagnosis and biopsy are crucial to ascertain the lesion's clinical behavior and any repercussions, leading to an accurate prognosis and diagnosis of the specific vascular abnormality for better treatment approaches. Therefore, it is important to diagnose and treat hemangiomas early.
